# Robot-Assisted Gastrectomy and Sigmoid Colectomy with Situs Inversus Totalis: A Case Report

**DOI:** 10.70352/scrj.cr.25-0073

**Published:** 2025-07-03

**Authors:** Midori Hara, Yoshinobu Ikeno, Junya Kobayashi, Daisuke Yagi, Hidemaro Yoshiba, Koji Doi, Toshiharu Aotake

**Affiliations:** Department of Gastrointestinal Surgery, Fukui Red Cross Hospital, Fukui, Fukui, Japan

**Keywords:** situs inversus totalis (SIT), robot assisted gastrectomy, robot assisted sigmoid colectomy

## Abstract

**INTRODUCTION:**

Situs inversus totalis is a rare congenital disorder defined as mirror-image transposition of the thoracoabdominal organs, and surgical strategies remain controversial owing to the associated anatomical abnormalities. Although laparoscopic and robot-assisted surgeries are gaining popularity, no reports have been published on robot-assisted gastrectomy and sigmoid colectomy for situs inversus totalis at the same time.

**CASE PRESENTATION:**

A 77-year-old woman with situs inversus totalis presented to our hospital with type 0-IIa+IIc sigmoid colon cancer and type 0-IIc gastric cancer. Therefore, we decided to perform robot-assisted gastrectomy and sigmoid colectomy. The port positions were the same for both procedures, and the robot rolled in from the opposite side of the body. The surgery was successful, and the postoperative course was uneventful.

**CONCLUSIONS:**

While we did not experience any challenges during distal gastrectomy, we experienced difficulty in dissecting toward the root of the inferior mesenteric artery during sigmoid colectomy. To address this problem, port transportation can be improved.

## Abbreviation

SIT
: situs inversus totalis

## INTRODUCTION

SIT is a rare congenital disorder defined as mirror-image transposition of the thoracoabdominal organs with an incidence of 1 in 20000 to 1 in 5000 individuals.^1)^ Owing to the reversed anatomy of the organs, the surgical approach for patients with SIT tends to be more complicated than usual, and surgeons must communicate well to adapt to the unfamiliar intraoperative images and techniques. Although laparoscopic and robot-assisted surgeries are gaining popularity, reports involving patients with SIT remain limited. Here, we report robot-assisted gastrectomy and sigmoid colectomy in a patient with SIT.

## CASE PRESENTATION

**Fig. 1:**
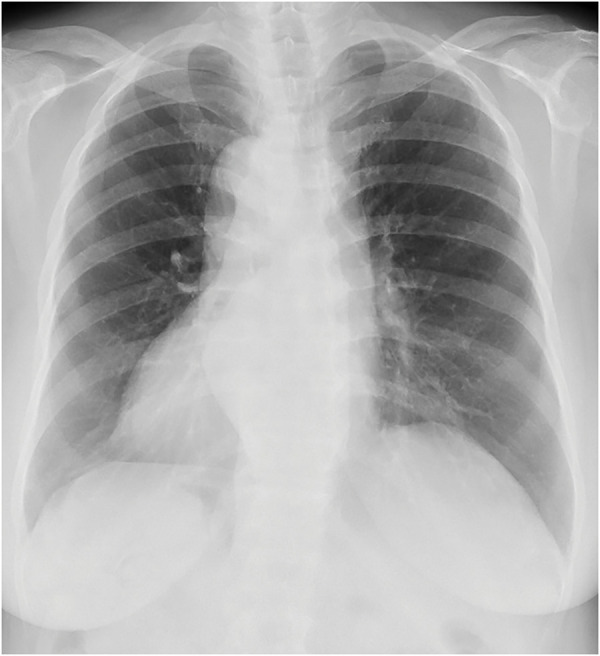
X-ray images of the patient. Dextrocardia is seen.

**Fig. 2:**
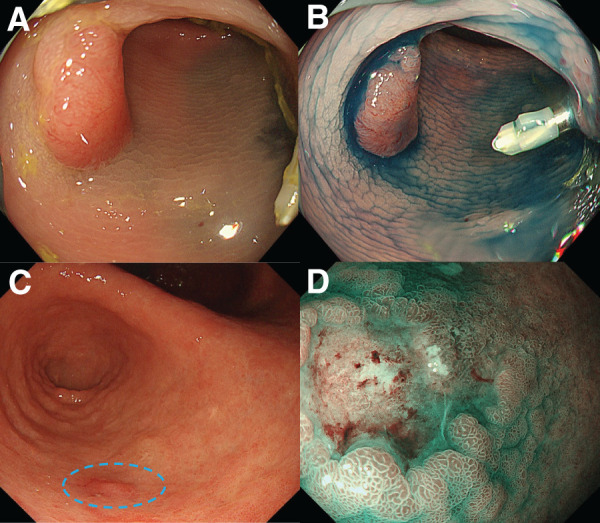
Endoscopy. (**A**, **B**) Lower gastrointestinal endoscopy revealed 0-IIa+IIc sigmoid colon cancer. (**C**, **D**) Upper gastrointestinal endoscopy revealed type 0-IIc gastric cancer located in the antrum of the greater curvature of the stomach.

**Fig. 3:**
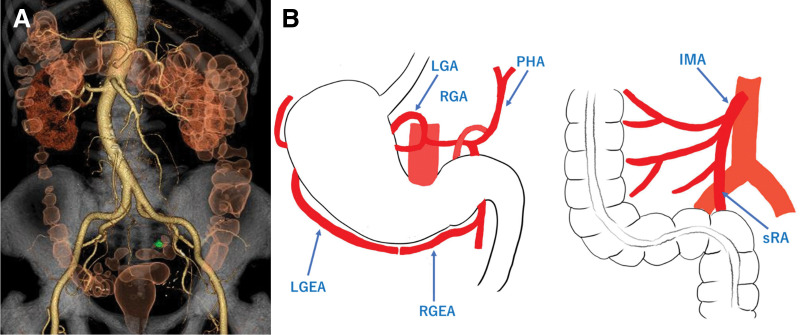
3D-CT. (**A**) 3D-CT revealed no vascular anomalies, except for complete inversion compared with the normal anatomy. (**B**) Schema shows arterial supply to stomach and sigmoid colon.

A 77-year-old woman who had been diagnosed with SIT by radiography (**Fig. 1**) presented to our hospital with suspected submucosal invasive sigmoid colon carcinoma. Lower gastrointestinal endoscopy revealed a type 0-IIa+IIc sigmoid colon cancer (**Fig. 2A, 2B**). CT did not show distant metastases but showed enlarged lymph nodes near the tumor. Upper gastrointestinal endoscopy revealed a type 0-IIc gastric cancer located in the antrum of the greater curvature of the stomach (**Fig. 2C, 2D**). 3D CT angiography revealed no vascular anomalies, except for complete inversion of the vessels compared with normal patients (**Fig. 3A, 3B**). The patient was 153.1 cm tall, weighed 64.0 kg, and had a preoperative body mass index of 27.3 kg/m^2^. Laboratory test results were within normal limits, except for carcinoembryonic antigen levels (328.5 ng/mL). Based on these findings, we elected to perform robot-assisted distal gastrectomy with Billroth II reconstruction and robot-assisted sigmoid colectomy simultaneously. Both procedures were conducted by the same surgeon, who has a license from the Japan Society for Endoscopic Surgery for both gastrectomy and colectomy using the da Vinci Xi Surgical System (Intuitive Surgical, Sunnyvale, CA, USA).

**Fig. 4:**
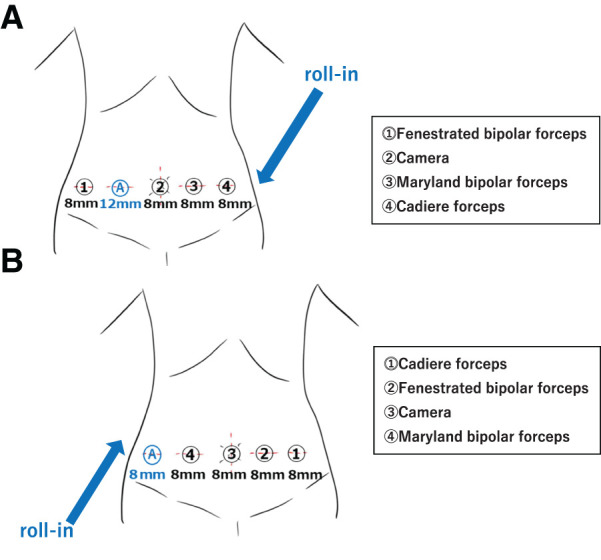
Trocar and arm position settings of the da Vinci surgical system. (**A**) The da Vinci system was rolled in from the left cranial side of the patient during distal gastrectomy. (**B**) The da Vinci system was rolled in from the caudal left side of the patient for the sigmoid colectomy.

**Fig. 5:**
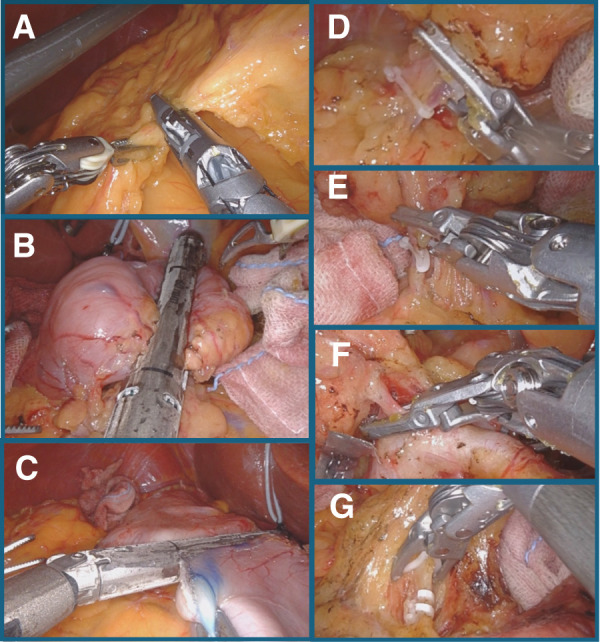
Intraoperative findings of a gastrectomy. (**A**) Dissection of the greater omentum was easy because its direction was toward the right side of the body. (**B**) The duodenum was dissected by the Endo-GIA purple 60 mm stapler. (**C**) The stomach was dissected twice using the Endo-GIA purple 60 mm stapler. (**D**) The left gastroepiploic artery, located on the right side of the body, was divided. (**E**) The right gastroepiploic artery, located on the left side, was identified and divided. (**F**) The right gastric artery, located on the left side and bifurcating from the proper hepatic artery, was confirmed and divided. (**G**) The left gastric artery, which was located on the right side, was divided.

First, we performed a distal gastrectomy. The robot was rolled in from the left side of the patient, and its instrument port positions are shown in **Fig. 4A**. The patient was placed in the supine position with her head positioned 10° downward and 5° right-upward. The 2nd arm was docked to the 30° endoscope, 1st arm to the fenestrated bipolar forceps, 3rd arm to the Maryland bipolar forceps, and 4th arm to the Cadiere forceps (**Fig. 4A**). The surgeon controlled the 3rd and 4th arms with the right hand, and the 1st arm with the left hand. The greater omentum was dissected along the border of the transverse colon from the middle to the right side (**Fig. 5A**) and the left gastroepiploic artery, located on the right side of the body, was divided (**Fig. 5D**). The right gastroepiploic artery, located on the left side and bifurcating from the gastroduodenal artery, was identified and divided (**Fig. 5E**). The duodenum was dissected by a tri-staple curved tip purple 60 mm cartridge (Endo GIA 60; Medtronic, Dublin, Ireland) (**Fig. 5B**). Subsequently, the lesser omentum was dissected. The right gastric artery, located on the left side and bifurcating from the proper hepatic artery, was confirmed and divided (**Fig. 5F**). The common hepatic artery was then exposed along the plane of the periarterial plexus and coronary vein, and the left gastric artery, which was located on the right side, was divided (**Fig. 5G**). After marking the tumor site, the stomach was dissected twice using the Endo-GIA purple 60 mm stapler and reconstructed using the Billroth II method (**Fig. 5C**).

**Fig. 6:**
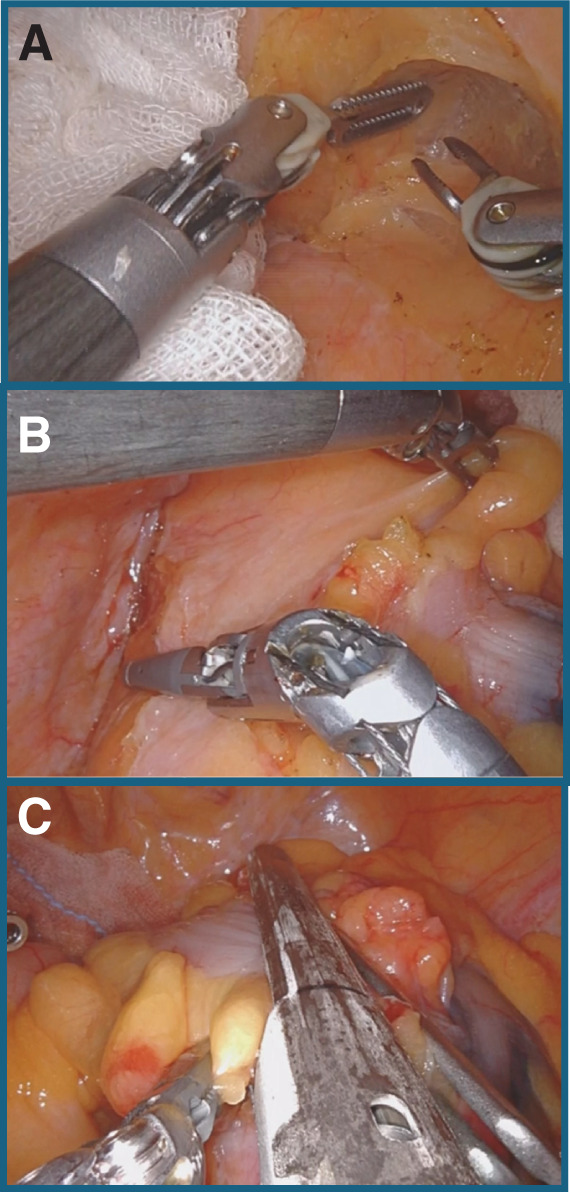
Intraoperative findings of a sigmoid colectomy. (**A**) Rectal mesenteric treatment was performed. (**B**) During sigmoid colectomy, dissection of the dorsal side of the inferior mesenteric artery toward the left caused some awkwardness, as the bending angle of the energy device was difficult to handle. (**C**) The anal side was divided by an Endo-GIA purple 60 mm stapler 6 cm from the peritoneal reflection.

Second, a sigmoid colectomy was performed. The robot was rolled in from the left side of the patient; the instrument port positions are shown in **Fig. 4B**. The patient was placed in the supine position with her head positioned 12° downward and 10° right-upward. We placed the da Vinci 8 mm port into a 12 mm assist port for the distal gastrectomy. The surgeon controlled the 4th arm on the right and the 1st and 2nd arms on the left side. The sigmoid colon and rectum were mobilized using a medial approach. The tumor was identified 10 cm from the peritoneal reflection. Dissection of the dorsal side of the inferior mesenteric artery proceeded toward the cranial side, which was opposite to the normal dissection (**Fig. 6A**), and the inferior mesenteric artery was identified and divided. After rectal mesenteric treatment was performed (**Fig. 6B**), the anal side was divided by an Endo-GIA purple 60 mm stapler 6 cm from the peritoneal reflection (**Fig. 6C**). The pneumoperitoneum was stopped, and the proximal side was divided. After the pneumoperitoneum was restored, anastomosis was completed by a Powered Circular stapler 25 mm (Echelon Circular Powered Stapler; Ethicon, Cincinnati, OH, USA). A drainage tube was placed on the dorsal side of the anastomosis from the lower left abdomen as our routine manner. Both gastric and colonic specimens were extracted from the approximately 5-cm navel wound.

The operation time was 535 min with 70.9 mL blood loss. The postoperative course was uneventful, and the patient was discharged on the 11th postoperative day. Pathological specimens were classified as pT1bN1bM0, pStage1b for gastric cancer and pT1bN1bM0, pStage3a for sigmoid colon cancer.

## DISCUSSION

SIT is a rare congenital disorder in which the inhibition of extraembryonic fluid flow during embryogenesis is associated with mirror-image anatomy. Surgery in patients with SIT is considered more difficult. Patients with SIT have a higher incidence of abnormal vascular variations.^2)^ Therefore, 3D-CT and CT angiography are useful for clarifying anatomy. Although our patient did not have vascular abnormalities, except for the vessels being a mirror-image of the normal anatomy, the imaging was useful for us to understand the anatomy and develop a strategy for surgery.

Minimally invasive surgery has been gaining popularity recently, and reports of both robot-assisted and laparoscopic surgeries for SIT can be found. Although in most laparoscopic surgeries the surgeon and assistant change their positions to avoid mirror images, right-handed surgeons encounter technical difficulties when using an energy device with their left hand. If they do not switch their positions, they need to adjust to the mirror image.^3,4)^ However, these challenges can be overcome by robot-assisted surgery, in which multi-joint forceps allow surgeons to flexibly manipulate and use energy devices with their dominant hands. In addition, tremor filtration facilitates smooth manipulation during surgery even with the non-dominant hand. These advantages improve surgical safety and aid complicated surgery for any anatomical anomaly.

Before surgery, we discussed whether to perform gastrectomy and sigmoid colectomy separately or simultaneously. We were able to find several reports of robot-assisted surgery for gastric cancer and rectal cancer in patients with SIT as shown in **Tables 1**^5–8)^ and **2**.^9–11)^ Based on the operating time and complications in these reports, we determined that performing both surgeries simultaneously was the better choice. Furthermore, considering the patient’s age, we determined that a single session of general anesthesia and surgery would be the most appropriate. Although the port positions and procedures differed in each report, we decided to use the same port for both surgeries, which was set on a horizontal line (**Fig. 4A, 4B**). Owing to this, we were able to create minimal wounds, and, in our procedure, the anatomical understanding was clear because the surgical view only required a mirror image of the horizontal. However, during the surgery, several issues relating to manipulation arose. During the gastrectomy, it was easy to dissect the greater omentum because its direction of dissection was toward the cranial side of the body, and using energy devices with the right hand was comfortable. However, during the sigmoid colectomy, there was some awkwardness with manipulation using the right hand when we dissected the dorsal side of the inferior mesenteric artery toward the cranial side, which was opposite to the usual medial approach. This is because the sigmoid colon was located on the right side of the body, whereas the stomach was located near the center. Port placement can be improved to overcome this difficulty. First, switching the camera to the 2nd arm enables the surgeon to manipulate the 3rd arm (Maryland) and 4th arm with the right hand, and dissection is easier. Second, placing the 3rd port on the slightly caudal side allows the 3rd arm to be located on the caudal side of the root of the inferior mesenteric artery during the entire dissection.

**Table 1:** Reports of robot assisted surgery for distal gastrectomy in patients with SIT

Author	Year	Operative time (min)	Blood loss (mL)	Postsurgical complications
Abbey et al.^5)^	2021	205	20	None
Ojima et al.^6)^	2019	260	20	None
Alhossaini et al.^7)^	2017	195	30	None
Kim et al.^8)^	2012	300	No data	None

**Table 2:** Reports of robot assisted surgery for rectal colectomy in patients with SIT

Author	Year	Operative time (min)	Blood loss (mL)	Postsurgical complications
Kasai et al.^11)^	2021	194	a little	None
Cui et al.^10)^	2018	210	50	None
Foo et al.^9)^	2015	204	100	None

## CONCLUSIONS

We performed robot-assisted gastrectomy and sigmoid colectomy for a patient with SIT and achieved successful results. Developing a detailed strategy to adapt to the abnormal anatomy is vital prior to the surgery. To standardize the surgical technique for SIT, there is a need to accumulate and analyze successful cases.

## DECLARATIONS

### Funding

None.

### Authors’ contributions

MH drafted the manuscript.

YI revised the manuscript.

All the authors approved the final manuscript.

### Availability of data and materials

Data supporting the findings of this study are available from the corresponding author upon reasonable request.

### Ethics approval and consent to participate

Not applicable.

### Consent for publication

Consent for publication was obtained from the patient.

### Competing interests

No competing financial interests exist.
